# Prognostic Nutritional Index Predicts In-Hospital Mortality Among Patients with *Clostridioides difficile* Infection: A Real-World Retrospective Study

**DOI:** 10.3390/antibiotics15070650

**Published:** 2026-06-30

**Authors:** Edison Jahaj, Dimitris C. Kounatidis, Eleni Mylona, Fotis Panagopoulos, Andreas Adamou, Sofia Kargioti, Maria Masouridi, Natalia G. Vallianou

**Affiliations:** 1 Department of Dermatology, Evangelismos General Hospital, 10676 Athens, Greece; 2 First Department of Internal Medicine, Sismanogleio General Hospital, 15126 Athens, Greece; 3 Department of Pharmacology, National and Kapodistrian University of Athens, 11527 Athens, Greece; 4 Fifth Department of Internal Medicine and Infectious Diseases, Evaggelismos General Hospital, 10676 Athens, Greece; 5 Infection Control Committee, Sismanogleio General Hospital, 15126 Athens, Greece

**Keywords:** *Clostridioides difficile* infection, inflammatory indices, in-hospital mortality, nutritional status, prognostic nutritional index, serum albumin

## Abstract

**Background/Objectives:** *Clostridioides difficile* infection (CDI) remains a major cause of morbidity and mortality, particularly among hospitalized older adults. This study evaluated the prognostic performance of routinely available inflammatory and nutritional biomarkers for predicting in-hospital mortality in patients with CDI. **Methods:** We conducted a retrospective observational study of 110 adults with confirmed CDI admitted to the Internal Medicine Department of a tertiary-care hospital in Athens, Greece, between January 2022 and December 2025. Demographic, clinical, and laboratory data obtained within 24 h of admission were analyzed. The prognostic value of the neutrophil-to-lymphocyte ratio (NLR), derived neutrophil-to-lymphocyte ratio (dNLR), C-reactive protein-to-albumin ratio (CAR), and Prognostic Nutritional Index (PNI) was assessed using univariate and multivariate logistic regression analyses. Receiver operating characteristic (ROC) curve analysis evaluated the discriminatory performance of PNI. **Results:** Twenty-two patients (20.0%) died during hospitalization. Compared with survivors, non-survivors exhibited significantly higher NLR (*p* = 0.035), dNLR (*p* = 0.012), and CAR (*p* = 0.015) values, whereas serum albumin and PNI were significantly lower (both *p* < 0.001). In univariate analysis, dNLR, CAR, and PNI were associated with mortality. After adjustment for age, sex, length of stay, and cancer diagnosis, only PNI remained independently associated with in-hospital mortality (*p* = 0.018). PNI showed good predictive performance (*p* < 0.001). **Conclusions:** PNI is a simple, inexpensive, and readily obtainable biomarker independently associated with in-hospital mortality in CDI. These findings highlight the importance of immune-nutritional status in CDI and support the potential utility of PNI for early risk stratification in hospitalized patients.

## 1. Introduction

*Clostridioides difficile* infection (CDI) remains a major public health concern and one of the leading causes of healthcare-associated infectious diarrhea worldwide. Although traditionally regarded as a predominantly nosocomial infection, the epidemiology of CDI has evolved considerably [1,2]. Community-acquired CDI (CA-CDI) now accounts for an increasing proportion of cases, representing nearly half of all CDI diagnoses in the United States according to data from the Centers for Disease Control and Prevention (CDC) [3,4]. Furthermore, recent evidence suggests that the transmission dynamics of CDI extend beyond direct hospital spread. De-la-Rosa-Martinez et al. reported that in-hospital transmission may account for only a limited proportion of cases, whereas asymptomatic colonization among hospitalized individuals may serve as an important reservoir for subsequent community dissemination following discharge [5]. These observations underscore the need for improved strategies aimed at reducing the overall burden of CDI across both healthcare and community settings.

The clinical manifestations of CDI range from mild, self-limited diarrhea to fulminant colitis associated with substantial morbidity and mortality. Severe disease may result in life-threatening complications, including septic shock, ileus, toxic megacolon, bowel perforation, and multiorgan failure [6]. Current guidelines from the American College of Gastroenterologists define severe CDI by the presence of a white blood cell count (WBC) > 15,000 cells/mm^3^ and/or a serum creatinine level ≥ 1.5 mg/dL [7]. Although these parameters are useful for assessing disease severity at presentation, they do not fully capture the complexity of host-related factors that influence clinical outcomes. Consequently, the identification of reliable, inexpensive, and readily available biomarkers capable of predicting adverse outcomes, particularly in-hospital mortality, remains an important unmet clinical need.

Increasing evidence suggests that systemic inflammation and nutritional status are important determinants of clinical outcomes across a broad spectrum of disorders, including infectious diseases, cardiometabolic diseases, and malignancy [8,9,10]. In this context, several biomarkers derived from routine laboratory assessment have emerged as promising prognostic tools due to their potential to reflect different aspects of the host response, including immune competence and nutritional reserve. Among the most extensively investigated are the neutrophil-to-lymphocyte ratio (NLR), derived neutrophil-to-lymphocyte ratio (dNLR), the platelet-to-neutrophil ratio (PNR), the systemic immune-inflammation index (SII), C-reactive protein-to-albumin ratio (CAR), serum albumin, and the Prognostic Nutritional Index (PNI) [10,11,12]. PNI is calculated using the following formula: 10 × serum albumin (g/dL) + 0.005 × total lymphocyte count (/μL). Likewise, a growing body of evidence has linked NLR, dNLR, CAR, and other inflammatory biomarkers to disease severity, complications, and mortality in CDI [13,14]. In contrast, evidence regarding the prognostic significance of PNI in CDI remains scarce [15].

The present retrospective study aimed to investigate the association between routinely available inflammatory and nutritional biomarkers and in-hospital mortality among patients hospitalized with CDI. By evaluating these readily accessible indices in a real-world clinical setting, we sought to identify practical tools that could facilitate early risk stratification and improve prognostic assessment in this vulnerable patient population.

## 2. Results

### 2.1. Demographic, Clinical and Baseline Laboratory Characteristics of the Study Population

A total of 110 patients with confirmed CDI were included in the study. The cohort comprised 53 female patients (48.2%), and the median age was 85 years (IQR: 75–89). Overall, 22 patients (20.0%) died during hospitalization, while 88 patients (80.0%) survived and were discharged. Patients were stratified into survivors and non-survivors according to in-hospital mortality. Demographic characteristics, comorbidities, and laboratory findings obtained within the first 24 h of admission are summarized in Table 1.

No significant differences were observed between survivors and non-survivors regarding age, sex, length of hospital stay (LOS), or the prevalence of major comorbidities, including malignancy, diabetes, heart failure, liver insufficiency, chronic kidney disease (CKD), and respiratory failure (all *p* > 0.05).

Among hematological parameters, non-survivors exhibited significantly lower lymphocyte counts compared with survivors [0.87 (0.59–1.40) vs. 1.30 (0.89–1.82) ×10^3^/μL, *p* = 0.013]. In contrast, total WBC counts, neutrophil counts, platelet counts, hemoglobin levels, and other leukocyte subpopulations did not differ significantly between the two groups. Several inflammatory and immune-nutritional biomarkers demonstrated significant associations with in-hospital mortality. Non-survivors presented significantly higher NLR values [12.57 (5.67–25.80) vs. 7.06 (3.87–16.81), *p* = 0.035], dNLR values [7.36 (3.74–12.51) vs. 4.03 (2.39–7.50), *p* = 0.012], and CAR values [30.99 (11.85–70.47) vs. 20.33 (7.91–36.42), *p* = 0.015] compared with survivors. Conversely, serum albumin concentrations [2.60 (2.38–2.90) vs. 3.20 (2.70–3.70) g/dL, *p* < 0.001] and PNI values [30.40 (28.66–35.08) vs. 39.28 (33.83–43.65), *p* < 0.001] were significantly lower among patients who died during hospitalization.

Among biochemical parameters, troponin concentrations were significantly higher in non-survivors than in survivors [32.00 (21.00–56.50) vs. 14.00 (7.00–38.00) ng/dL, *p* = 0.006]. No significant between-group differences were observed for renal function indices, liver biochemistry, glucose levels, total protein, lactate dehydrogenase (LDH), creatine phosphokinase (CPK), or the remaining biochemical parameters evaluated. Overall, the most pronounced differences between survivors and non-survivors were observed for serum albumin, PNI, dNLR, NLR, CAR, lymphocyte count, and troponin levels.

### 2.2. Association Between Prognostic Biomarkers and In-Hospital Mortality

To further investigate the relationship between inflammatory and nutritional biomarkers and mortality, univariate and multivariate logistic regression analyses were performed. The evaluated variables included NLR, dNLR, CAR, PNI, age, sex, LOS, and the presence of malignancy. The results of both models are presented in Table 2.

In the univariate analysis, higher dNLR and CAR values were associated with increased odds of in-hospital mortality. Specifically, each one-unit increase in dNLR was associated with an 11.2% increase in mortality risk (OR: 1.112, 95% CI: 1.013–1.220, *p* = 0.025), while each one-unit increase in CAR was associated with a 2.5% increase in mortality risk (OR: 1.025, 95% CI: 1.007–1.043, *p* = 0.006). In contrast, PNI demonstrated a significant protective effect, with higher values associated with lower mortality risk (OR: 0.884, 95% CI: 0.822–0.952, *p* = 0.001).

A multivariate logistic regression model was subsequently constructed to account for potential confounding factors. After adjustment, PNI remained the only biomarker independently associated with in-hospital mortality. Specifically, each one-unit increase in PNI was associated with a 12% reduction in the odds of death during hospitalization (OR: 0.883, 95% CI: 0.796–0.979, *p* = 0.018). Neither dNLR nor CAR retained statistical significance in the adjusted model. Notably, age, sex, LOS, and the presence of malignancy were not independently associated with mortality in either univariate or multivariate analyses.

### 2.3. Prognostic Performance of the Prognostic Nutritional Index

**Figure 1:**
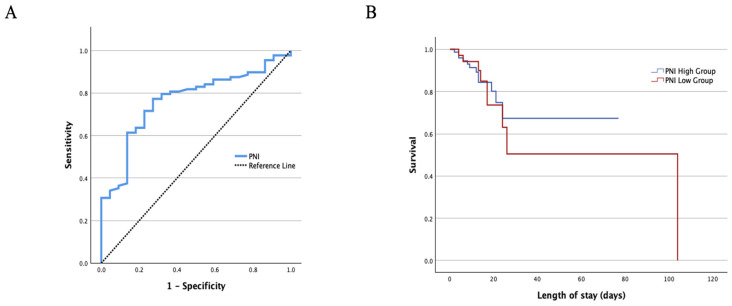
Diagnostic performance and survival analysis of PNI. (**A**) A receiver operating characteristic (ROC) curve was generated for PNI (blue line), demonstrating its prognostic value for mortality. PNI presented an AUC equal to 0.766 (95% CI: 0.666–0.8660; *p* < 0.001) with a cut-off value of 33.3, calculated based on the Youden’s Index. The selected cut-off value corresponds to 77.3% sensitivity and 72.7% specificity. (**B**) An exploratory Kaplan–Meier analysis was performed to evaluate the probability for mortality in correlation to PNI. The log-rank test was used for two-group comparisons. The cohort was dichotomized above and below the determined cut-off value (33.3; PNI high group and PNI low group). Mean survival time for the PNI high group (blue line) was 57 ± 6 days, whereas for the PNI low group (red line) was 62 ± 13 days (log-rank test, *p* = 0.69). Abbreviations: AUC: area under the curve; CI: confidence interval; PNI: prognostic nutritional index.

Given its independent association with mortality, the prognostic performance of PNI was further evaluated using receiver operating characteristic (ROC) curve analysis (Figure 1A). PNI demonstrated good discriminatory ability for predicting in-hospital mortality, yielding an area under the curve (AUC) of 0.766 (95% CI: 0.666–0.866, *p* < 0.001). The optimal cut-off value, determined using Youden’s index, was 33.3. This threshold provided a sensitivity of 77.3% and a specificity of 72.7% for predicting mortality. Patients with PNI values below 33.3 were more likely to experience in-hospital death, whereas values above this threshold were associated with survival. To further explore the clinical utility of this cut-off, Kaplan–Meier survival analysis was performed after stratifying patients into low-PNI (<33.3) and high-PNI (≥33.3) groups. This Kaplan–Meier analysis was performed as an exploratory sensitivity analysis to determine whether the ROC-derived PNI cutoff was associated with survival probability over time; the absence of a significant between-group difference indicates that this cutoff should be considered preliminary (Figure 1B).

Throughout the observation period, cumulative survival remained above 50% in both groups, precluding calculation of median survival times. Mean survival time was 57 ± 6 days in the high-PNI group and 62 ± 13 days in the low-PNI group. However, no statistically significant difference in survival probability was observed between the two groups according to the log-rank test (*p* = 0.69).

## 3. Discussion

In our study, the mortality rate was 20%. It should be pointed out that our study population was confined to elderly people, amongst whom mortality rates tend to be increased due to malnutrition, comorbidities and the aging process [3,4]. However, it is noteworthy that allcause mortality rates within 30 days of CDI have been estimated to approach 20%, whereas mortality due to CDI ranges between 5% and 7% [3,4].

The principal finding of the present study is that PNI was independently associated with in-hospital mortality among patients with CDI. Although several inflammatory biomarkers, including NLR, dNLR, and CAR, differed significantly between survivors and non-survivors in univariate analyses, only PNI retained statistical significance after adjustment for potential confounders. Furthermore, PNI demonstrated good discriminatory performance for mortality prediction, highlighting the potential importance of immune-nutritional status in determining clinical outcomes among patients with CDI.

PNI is a composite index derived from serum albumin concentration and peripheral lymphocyte count. Thus, it provides an integrated assessment of both nutritional status and immune competence [16]. Serum albumin is traditionally regarded as a marker of nutritional status; however, it is also a well-established negative acute-phase reactant. During systemic inflammation, pro-inflammatory cytokines suppress hepatic albumin synthesis and increase vascular permeability, resulting in reduced circulating albumin levels [17,18,19]. Consequently, hypoalbuminemia may reflect not only malnutrition but also the magnitude of the inflammatory response. At the same time, lymphocyte count represents an important component of host immune function. Lymphocytes play a central role in adaptive immunity and are critically involved in both the early and late phases of the immune response to severe infection. Lymphopenia is a common feature of sepsis and has been associated with immune dysregulation and adverse clinical outcomes [20]. By combining these two parameters, PNI may provide a more comprehensive assessment of the host’s immune-nutritional reserve than either variable alone [16]. Although this study did not directly test the incremental prognostic value of PNI over albumin alone, PNI may offer a biologically meaningful composite assessment by integrating hypoalbuminemia with lymphopenia, two markers relevant to inflammation, nutritional reserve, and immune competence.

Recent evidence supports the prognostic value of PNI across a broad spectrum of chronic diseases in which inflammation plays a central role in pathophysiology. Multiple studies have reported associations between lower PNI values and adverse clinical outcomes, particularly in cardiometabolic disorders and CKD [21,22,23]. For example, analyses of data from the National Health and Nutrition Examination Survey (NHANES) demonstrated that lower PNI levels were associated with increased risks of all-cause and cardiovascular mortality among individuals with type 2 diabetes (T2D) and prediabetes [24]. In addition, PNI has been proposed as a potential marker reflecting the achievement of key therapeutic targets in T2D management, including glycemic control, blood pressure regulation, lipid management, and weight control [25]. Emerging evidence further suggests a role for PNI in identifying patients at increased risk of diabetes-related cardiovascular complications, including acute coronary syndrome and heart failure-related outcomes [26,27]. Interestingly, PNI has also been investigated as a marker of metabolic dysfunction-associated steatotic liver disease (MASLD), where lower values have been associated with a greater disease burden in a dose-dependent manner, particularly among younger individuals without hyperlipidemia or hyperglycemia [22].

Sato et al. followed 1046 adults with CKD risk factors, preserved renal function, and no proteinuria and found that PNI was associated with the subsequent development of CKD [28]. Similarly, Yu et al. reported that lower PNI values were associated with future renal function decline in patients with T2D and preserved renal function and suggested that the combination of PNI and hemoglobin A1c (HbA1c) may improve risk stratification [29]. Moreover, lower PNI values have been associated with increased risks of cardiovascular and all-cause mortality among patients with CKD [30], while PNI may provide additional prognostic information beyond established cardiovascular risk factors in this population [22]. It has been suggested that the cut-off of 33.3 for PNI has been designated to be related to higher risk, whereas levels of PNI above 42 have been associated with better prognosis. The ranges between 33.3 and 42 have been linked to an intermediate risk [31]. Collectively, these findings suggest that PNI reflects not only acute inflammatory and nutritional disturbances but also the cumulative burden of chronic disease and impaired physiological reserve.

Additional support for this concept comes from studies conducted in other clinical settings. Kiryat et al. reported that lower PNI values were associated with greater disease severity and adverse outcomes among patients presenting to the emergency department with gastrointestinal emergencies. Notably, patients with conditions commonly linked to chronic nutritional impairment, including cirrhosis and malignancy, exhibited higher risks of hospitalization and intensive care unit (ICU) admission [31]. Similarly, recent oncological data have shown that lower PNI values are associated with reduced skeletal muscle and adipose tissue mass, as well as poorer survival outcomes in patients with oligometastatic non-small cell lung carcinoma (NSCLC) undergoing radiotherapy [32].

The prognostic value of PNI has also been explored in infectious diseases. In a large retrospective study including more than 32,000 patients with sepsis, Kyo et al. suggested that PNI may be useful for identifying patients at increased risk of mortality [33]. Furthermore, lower PNI values have been associated with an increased risk of Acute Respiratory Distress Syndrome (ARDS) among patients with sepsis, irrespective of diabetes status [34]. Outside the setting of sepsis, a recent comparative cohort analysis demonstrated that patients with concurrent coronavirus disease 2019 (COVID-19) and CDI exhibited higher NLR and CAR values and lower PNI values than patients with Severe Acute Respiratory Syndrome Coronavirus 2 (SARS-CoV-2) infection alone. In the same clinical setting, lower PNI values were associated with increased odds of ICU admission and mortality [15,35].

To the best of our knowledge, the present study is the first observational study specifically investigating the association between PNI and mortality in hospitalized patients with CDI in the absence of concomitant SARS-CoV-2 infection. We found that lower PNI values were independently associated with in-hospital mortality, even after adjustment for potential confounding factors. This association is biologically plausible, given that both malnutrition and immune dysfunction are highly prevalent among older adults, who constitute the population most frequently affected by CDI.

An increasing number of studies have suggested a prognostic role for inflammatory indices such as NLR, dNLR, hs-CRP, serum albumin, and CAR in assessing CDI severity and outcomes, both in patients with and without major comorbidities [36,37,38,39,40]. Consistent with previous reports, we observed significant differences in NLR, dNLR, serum albumin, and CAR between survivors and non-survivors. However, these associations did not persist after multivariable adjustment, with PNI emerging as the only independent predictor of mortality. This finding may be explained, at least in part, by the relatively limited sample size and number of mortality events, which may have reduced the statistical power required to detect independent associations for the remaining biomarkers. Indeed, in a previous epidemiological study of 274 hospitalized patients with CDI, we observed significant associations between mortality and several inflammatory biomarkers, including NLR, dNLR, and hs-CRP [40].

The present study has several limitations. First, its retrospective single-center design introduces the possibility of selection bias and limits the generalizability of the findings. Second, the relatively small sample size and limited number of mortality events may have reduced statistical power and contributed to the loss of significance observed for certain biomarkers in multivariable analyses. Third, laboratory parameters were assessed only at admission, precluding evaluation of dynamic changes during hospitalization. Finally, external validation in independent cohorts was not available. Despite these limitations, our study has important strengths. We evaluated a real-world cohort of hospitalized patients with confirmed CDI and focused on biomarkers that are inexpensive, routinely available, and easily applicable in everyday clinical practice. The identification of PNI as an independent predictor of mortality suggests that assessment of immune-nutritional status may provide clinically meaningful prognostic information beyond traditional inflammatory markers.

Future large-scale prospective studies are warranted to validate these findings, establish optimal PNI cut-off values, and determine whether incorporation of PNI into existing severity assessment tools could improve risk stratification and clinical decision-making in patients with CDI. In particular, further investigation is needed to clarify whether PNI may predict not only mortality but also overall disease severity, ICU admission, recurrence, and other clinically relevant outcomes. Additionally, it remains to be elucidated whether nutritional interventions and Medical Nutritional Therapy (MNT) could play a role in mitigating mortality rates among patients with CDI. MNT strategies could have an impact in this regard. Thus, further studies are eagerly anticipated in order to assess whether MNT could play a pivotal role in reducing mortality among patients with CDI.

## 4. Materials and Methods

### 4.1. Study Design and Patient Population

This retrospective study included 110 consecutive adult patients with confirmed CDI who were hospitalized in the First Department of Internal Medicine at Sismanogleio General Hospital, a tertiary-care hospital in Athens, Greece, between 1 January 2022 and 31 December 2025. CDI was defined as the presence of compatible clinical symptoms, such as diarrhea, in conjunction with a positive laboratory testing for *Clostridioides difficile* toxin A and/or toxin B.

The study was conducted in accordance with the ethical principles of the Declaration of Helsinki, as revised in 2013. Ethical approval was obtained from the Institutional Review Board of Sismanogleio General Hospital (Protocol No. 4165/18 February 2025).

### 4.2. Data Collection and Laboratory Parameters

Demographic and clinical data were retrieved from electronic medical records. The following variables were recorded: age, sex, LOS, in-hospital outcome (survivors versus non-survivors), and the presence of major comorbidities, including malignancy, diabetes, CKD, chronic respiratory failure, and liver insufficiency. Laboratory parameters obtained within the first 24 h of admission included WBC, hematocrit, hemoglobin, platelet count, high-sensitivity C-reactive protein (hs-CRP), serum albumin, total serum protein, bilirubin, creatinine, glucose, urea, aspartate aminotransferase (AST), alanine aminotransferase (ALT), alkaline phosphatase (ALP), gamma-glutamyl transferase (GGT), LDH, and cardiac troponin levels.

Several inflammatory and immune-nutritional indices were calculated using standard formulas: (i). NLR = neutrophil count/lymphocyte count; (ii). dNLR = neutrophil count/WBC − neutrophil count; (iii). Platelet-to-neutrophil ratio (PNR) = platelet count/neutrophil count; (iv). Platelet-to-lymphocyte ratio (PLR) = platelet count/lymphocyte count; (v). CAR = CRP/albumin; (vi). Systemic Immune-Inflammation Index (SII) = platelet count × neutrophil count/lymphocyte count, and PNI = 10 × serum albumin (g/dL) + 0.005 × total lymphocyte count (/μL)]. In addition, the estimated glomerular filtration rate (eGFR) was calculated using the Chronic Kidney Disease Epidemiology Collaboration (CKD-EPI) equation.

## 5. Statistical Analysis

Categorical variables are presented as absolute numbers and percentages, whereas continuous variables are expressed as mean ± standard deviation (SD) or median with interquartile range (IQR), as appropriate. The distribution of continuous variables was assessed using the Shapiro–Wilk test. Comparisons between categorical variables were performed using the chi-square test. For continuous variables, comparisons between two groups were conducted using the independent-samples Student’s *t*-test for normally distributed data or the Mann–Whitney *U* test for non-normally distributed variables.

To identify factors associated with in-hospital mortality, univariate logistic regression analyses were initially performed. Variables considered clinically relevant or demonstrating statistical significance in univariate analysis were subsequently entered into a multivariate logistic regression model. Results are reported as OR with corresponding 95% CI. ROC curve analysis was performed to evaluate the discriminatory ability of the investigated biomarkers for predicting in-hospital mortality. AUC was calculated together with its 95% CI. Optimal cut-off values were determined using Youden’s index, and the corresponding sensitivity and specificity values were reported.

All statistical tests were two-sided, and a *p*-value < 0.05 was considered statistically significant. Statistical analyses were performed using IBM SPSS Statistics version 30.0 (IBM Corp., Armonk, NY, USA) and the R statistical software environment (R Foundation for Statistical Computing, Vienna, Austria).

## 6. Conclusions

This retrospective study demonstrates that PNI is independently associated with in-hospital mortality among patients hospitalized with CDI. Among several inflammatory and nutritional biomarkers evaluated, PNI emerged as the only independent predictor of mortality after multivariable adjustment and showed good discriminatory performance in ROC analysis. These findings highlight the importance of immune-nutritional status in CDI and suggest that PNI may represent a simple, inexpensive, and readily available tool for early risk stratification in hospitalized patients. Further large-scale prospective studies are needed to validate its clinical utility and define its role within prognostic algorithms for CDI.

## Figures and Tables

**Table 1:** Demographic, clinical, and baseline laboratory characteristics of patients with CDI stratified by in-hospital mortality.

Variable	Survivors (*n* = 88)	Non-Survivors (*n* = 22)	*p*-Value
Age (years), (median, IQR)	85.0 (75.0–89.8)	84.5 (76.8–89.3)	0.623
Sex, n (%)			0.445
Male, n (%)	44 (50.0)	13 (59.1)	
Female, n (%)	44 (50.0)	9 (40.9)	
Comorbidities, n (%)	67 (76.1)	20 (90.9)	0.128
Cancer, n (%)	14 (15.9)	5 (22.7)	0.449
Diabetes, n (%)	28 (31.8)	10 (45.5)	0.229
Heart failure, n (%)	32 (36.4)	8 (36.4)	>0.999
Liver insufficiency, n (%)	7 (8.0)	2 (9.1)	0.862
Renal failure, n (%)	27 (30.7)	11 (50.0)	0.088
Respiratory failure, n (%)	24 (27.3)	10 (45.5)	0.098
Length of stay (median, IQR)	13 (10–20)	13 (6–20)	0.507
Laboratory data (median, IQR)			
White blood cells (10^3^/μL)	11.07 (8.35–17.03)	11.73 (9.09–19.44)	0.575
Neutrophils (10^3^/μL)	8.74 (5.69–14.58)	10.56 (7.25–18.18)	0.372
Lymphocytes (10^3^/μL)	1.30 (0.89–1.82)	0.87 (0.59–1.40)	0.013 *
Monocytes (10^3^/μL)	0.70 (0.42–0.89)	0.55 (0.30–0.82)	0.120
Eosinophils (10^3^/μL)	0.04 (0–0.16)	0.03 (0–0.08)	0.297
Basophils (10^3^/μL)	0.04 (0.01–0.06)	0.03 (0.01–0.04)	0.058
Platelets (10^3^/μL)	299.0 (205.0–376.5)	250.0 (177.8–293.5)	0.068
Hematocrit (HCT) (%)	31.95 (27.68–35.98)	32.00 (23.90–36.23)	0.728
Hemoglobin (Hb) (g/dL)	10.60 (9.30–11.95)	10.05 (8.23–13.20)	0.943
NLR	7.06 (3.87–16.81)	12.57 (5.67–25.80)	0.035 *
dNLR	4.03 (2.39–7.50)	7.36 (3.74–12.51)	0.012 *
SII	1834 (943.5–4269)	2987 (1011–6480)	0.459
PNR	27.68 (17.00–49.07)	23.16 (14.14–30.15)	0.058
PLR	218.38 (159.13–333.62)	287.11 (179.10–366.43)	0.366
hs-CRP (mg/L)	65.80 (25.18–113.35)	84.65 (41.30–209.00)	0.073
Albumin (g/dL)	3.20 (2.70–3.70)	2.60 (2.38–2.90)	<0.001 ***
Total bilirubin (mg/dL)	0.54 (0.38–0.88)	0.61 (0.44–0.84)	0.562
Direct bilirubin (mg/dL)	0.24 (0.16–0.35)	0.30 (0.13–0.48)	0.413
PNI	39.28 (33.83–43.65)	30.40 (28.66–35.08)	<0.001 ***
CAR	20.33 (7.91–36.42)	30.99 (11.85–70.47)	0.015 *
Total protein (g/dL)	6.10 (5.28–6.73)	5.40 (4.90–6.55)	0.151
Glucose (mg/dL)	119.00 (99.00–144.00)	119.00 (95.25–205.75)	0.538
Troponin (ng/dL)	14.00 (7.00–38.00)	32.00 (21.00–56.50)	0.006 **
Urea (mg/dL)	52.00 (27.00–83.75)	53.00 (40.75–165.50)	0.054
Creatinine (mg/dL)	1.10 (0.80–1.88)	1.40 (0.88–2.90)	0.234
Aspartate aminotransferase (U/L)	18.00 (13.00–30.75)	18.50 (13.50–26.25)	0.920
Alkaline transaminase (U/L)	11.50 (7.25–18.00)	11.50 (6.75–18.25)	0.708
Alkaline phosphatase (U/L)	87.00 (72.00–106.75)	95.50 (72.25–130.75)	0.283
Gamma-glutamyltransferase (U/L)	18.50 (10.00–37.00)	16.00 (6.80–25.00)	0.233
Lactate dehydrogenase (U/L)	245.50 (189.00–348.00)	275.00 (236.25–351.25)	0.084
Creatine phosphokinase (U/L)	53.00 (30.00–114.00)	52.50 (29.50–104.25)	0.955
eGFR (mL/min/1.73 m^2^)	60.35 (31.60–81.85)	40.50 (20.00–66.50)	0.181

Data are presented as individual numbers, n (%), and median with interquartile range (IQR). Patients enrolled in the study cohort were assigned to one of two groups, survivors and non-survivors, based on in-hospital mortality. Two group comparisons for categorical variables were performed using the chi-square test. The Student’s *t*-test or the non-parametric Mann–Whitney *U* test was employed, as appropriate, for quantitative variables. Laboratory data were measured within 24 h of admission. Abbreviations: CAR: C-reactive protein to albumin ratio; dNLR: derived neutrophil-to-lymphocyte ratio; eGFR: estimated glomerular filtration rate; hs-CRP: high-sensitivity C-reactive protein; IQR: interquartile range; NLR: neutrophil-to-lymphocyte ratio; SII: systemic immune–inflammation index; PLR: platelet-to-lymphocyte ratio; PNI: prognostic nutritional index; PNR: platelet-to-neutrophil ratio. * *p*-value < 0.05; ** *p*-value < 0.01; *** *p*-value < 0.001.

**Table 2:** Results of the fitted univariate and multivariate regression models.

Variable	Univariate Model			Multivariate Model		
	OR	95% CI	*p*-Value	OR	95% CI	*p*-Value
NLR	1.027	0.994–1.061	0.111	0.973	0.910–1.041	0.433
dNLR	1.112	1.013–1.220	0.025 *	1.066	0.861–1.320	0.557
PNI	0.884	0.822–0.952	0.001 **	0.883	0.796–0.979	0.018 *
CAR	1.025	1.007–1.043	0.006 **	1.018	0.994–1.044	0.147
Age	1.022	0.981–1.065	0.297	1.009	0.959–1.062	0.725
Sex	0.692	0.269–1.785	0.447	0.721	0.226–2.306	0.581
LOS	1.003	0.972–1.036	0.836	1.014	0.980–1.049	0.419
Cancer	1.629	0.513–5.175	0.408	0.978	0.216–4.422	0.977

The association of NLR, dNLR, PNI, CAR, and age, sex, length of stay, and the positive diagnosis of cancer was evaluated in a univariate logistic regression model. A multivariate logistic regression model was fitted to adjust for confounders demonstrating PNI as the only independent indicator for mortality in patients with verified *Clostridioides difficile* infection. CAR: c-reactive protein to albumin ratio; CI: confidence interval; dNLR: derived neutrophil-to-lymphocyte ratio; LOS: length of stay; NLR: neutrophil-to-lymphocyte ratio; OR: odds ratio; PNI: prognostic nutritional index. *, *p*-value < 0.05; **, *p*-value < 0.01.

## Data Availability

Data is contained within the manuscript.

## Abbreviations

ARDS	Acute Respiratory Distress Syndrome
AUC	Area under the curve
CA-CDI	Community-acquired *Clostridioides difficile* infection
CAR	C-reactive protein-to-albumin ratio
CDI	*Clostridioides difficile* infection
CI	Confidence interval
CKD	Chronic kidney disease
CKD-EPI	Chronic Kidney Disease Epidemiology Collaboration
COVID-19	Coronavirus disease 2019
CPK	Creatine phosphokinase
dNLR	Derived neutrophil-to-lymphocyte ratio
eGFR	Estimated glomerular filtration rate
HbA1c	Hemoglobin A1c
hs-CRP	High-sensitivity C-reactive protein
ICU	Intensive care unit
IQR	Interquartile range
LDH	Lactate dehydrogenase
LOS	Length of hospital stay
MASLD	Metabolic dysfunction-associated steatotic liver disease
MNT	Medical Nutritional Therapy
NHANES	National Health and Nutrition Examination Survey
NLR	Neutrophil-to-lymphocyte ratio
NSCLC	Non-small cell lung carcinoma
OR	Odds ratio
PLR	Platelet-to-lymphocyte ratio
PNI	Prognostic Nutritional Index
PNR	Platelet-to-neutrophil ratio
ROC	Receiver operating characteristic
SARS-CoV-2	Severe Acute Respiratory Syndrome Coronavirus 2
SD	Standard deviation
SII	Systemic Immune-Inflammation Index
T2D	Type 2 diabetes
WBC	White blood cell
